# Supplementary material for: MetaQTL: a package of new computational methods for the meta-analysis of QTL mapping experiments
Source: BMC Bioinformatics. 2007 Feb 8;8:49. doi: 10.1186/1471-2105-8-49 (PMC1808479; doi:10.1186/1471-2105-8-49)
Supplement: Additional File 7 — MetaQTL Package : jar file and tutorial. This Zip archive contains both the MetaQTL JAR file and the files of the tutorial. [file 1471-2105-8-49-S7.zip › org.inra.metaqtl/doc/org/metaqtl/algo/HClustAlgorithm.html]

HClustAlgorithm


|  |  |  |
| --- | --- | --- |
| **Overview** \| **Package** \| **Class** \| **Use** \| **Tree** \| **Deprecated** \| **Index** \| **Help** | |  |
| **PREV CLASS**   **NEXT CLASS** | **FRAMES**    **NO FRAMES**     **All Classes** |  |
| SUMMARY: NESTED \| FIELD \| CONSTR \| METHOD | DETAIL: FIELD \| CONSTR \| METHOD |  |


---


## org.metaqtl.algo Class HClustAlgorithm

```
java.lang.Object
  org.metaqtl.algo.HClustAlgorithm
```

---

``` public class HClustAlgorithm extends java.lang.Object ```

This class implements stantard agglomerative hierarchical
clustering methods.

---

| **Field Summary** | |
| --- | --- |
| `static int` | `CENTROID_METHOD`             Centroid method |
| `static int` | `METHOD`             A global variable to control the custering method. |
| `static int` | `WARD_METHOD`             Ward method |


**Constructor Summary**

`HClustAlgorithm()`


| **Method Summary** | |
| --- | --- |
| `static Tree` | `CentroidMethod(double[] x, double[] sd)`             This method performs a hierarchical clustering of the given data points using a centroid method based on the mahalanobis distance. |
| `static Tree` | `run(double[] x, double[] sd)`             This method performs a agglomerative clustering of the given data points where `x` is the vector of observed values and `sd` the vector of their standard deviations. |
| `static Tree` | `WardMethod(double[] x, double[] sd)`             This method performs a hierarchical clustering of the given data points using the Ward's method. |

**Methods inherited from class java.lang.Object**

`clone, equals, finalize, getClass, hashCode, notify, notifyAll, toString, wait, wait, wait`

**Field Detail**

### CENTROID\_METHOD

```
public static final int CENTROID_METHOD
```

:   Centroid method

    **See Also:**: Constant Field Values

---


### WARD\_METHOD

```
public static final int WARD_METHOD
```

:   Ward method

    **See Also:**: Constant Field Values

---


### METHOD

```
public static int METHOD
```

:   A global variable to control the custering
    method.


**Constructor Detail**

### HClustAlgorithm

```
public HClustAlgorithm()
```


**Method Detail**

### run

```
public static Tree run(double[] x,
                       double[] sd)
```

:   This method performs a agglomerative clustering
    of the given data points where `x` is the
    vector of observed values and `sd` the
    vector of their standard deviations. It returns an
    array of `TreeNode` which represents the
    hierarchical clustering.

    :   **Parameters:**: `x` - the vector of data points.: `sd` - the vector of standard deviations. **Returns:**: the tree which represents the hierarchical clustering.

---


### CentroidMethod

```
public static Tree CentroidMethod(double[] x,
                                  double[] sd)
```

:   This method performs a hierarchical clustering
    of the given data points using a centroid method
    based on the mahalanobis distance.

    :   **Parameters:**: `x` - the vector of data points.: `sd` - the vector of standard deviations. **Returns:**: the tree which represents the hierarchical clustering.

---


### WardMethod

```
public static Tree WardMethod(double[] x,
                              double[] sd)
```

:   This method performs a hierarchical clustering
    of the given data points using the Ward's method.

    :   **Parameters:**: `x` - the vector of data points.: `sd` - the vector of standard deviations. **Returns:**: the tree which represents the hierarchical clustering.


---


|  |  |  |
| --- | --- | --- |
| **Overview** \| **Package** \| **Class** \| **Use** \| **Tree** \| **Deprecated** \| **Index** \| **Help** | |  |
| **PREV CLASS**   **NEXT CLASS** | **FRAMES**    **NO FRAMES**     **All Classes** |  |
| SUMMARY: NESTED \| FIELD \| CONSTR \| METHOD | DETAIL: FIELD \| CONSTR \| METHOD |  |


---
